# Differences in fixation to young and elderly quadrilateral surfaces with anatomic quadrilateral surface plate (AQSP) based on cortical thickness morphological results

**DOI:** 10.1186/s13018-022-03027-2

**Published:** 2022-03-05

**Authors:** Jialiang Guo, Weichong Dong, Yali Zhou, Jinglue Hu, Pengyu Ye, Wei Chen, Yingze Zhang, Zhiyong Hou

**Affiliations:** 1 The School of Medicine, Nankai University, Tianjin, People’s Republic of China; 2 Department of Orthopaedics, The Third Hospital of Hebei Medical University, Shijiazhuang, People’s Republic of China; 3 Department of Pharmacy, The Second Hospital of Hebei Medical University, Shijiazhuang, People’s Republic of China; 4 NHC Key Laboratory of Intelligent Orthopeadic Equipment (The Third Hospital of Hebei Medical University), Shijiazhuang, People’s Republic of China; 5 Chinese Academy of Engineering, Beijing, People’s Republic of China

**Keywords:** Cortical thickness, Quadrilateral surface, Anatomical quadrilateral surface plate, Acetabular fracture

## Abstract

**Aims:**

With relatively thinner cortical thickness, the management of acetabulum osteoporotic fractures in elderly patients is difficult. The aim of the research was to compare and present the morphological characteristics of the quadrilateral plate in young and elderly age groups, such as the area, and position distribution of the thin cortical thickness region, fracture lines maps, and propose a revised design plate for elderly patients based on these anatomic information.

**Methods:**

As a retrospective research, acetabular fracture with one normal hemipelvises, including 110 men and 39 women, were collected to present the morphological characteristics of the quadrilateral region. The subjects were divided into three different age groups: Group I = 18–40 years (31.3 ± 6.6 years), Group II = 41–60 years (49.9 ± 5.3 years), and Group III ≥ 61 years (68.7 ± 6.8 years). The area of the quadrilateral surface, the area and position distribution of the thin cortical thickness region, the ratio and fracture lines maps were calculated and compared with Mimics in different groups.

**Results:**

The thin cortical thickness/width region area (TCWRA) was significantly increased in Group III compared with Group I and Group II. The ratio of TCWRA accounted for in the quadrilateral region was also significantly increased in Group III (≥ 61 years) compared with Group I (*P* = 0.01) and Group II (*P* = 0.011). None of the subjects had a component involving the “A” zone, thirty-three thin cortical thickness regions were located in the “B” zone, and one hundred and sixteen involved both zones of the quadrilateral plate (“A + B” zone). Furthermore, there were a significant differences in the fracture line distributions in three age groups. More fracture lines of elderly patients were located at anterior part (B zone) compared with Group I and Group II.

**Conclusions:**

It was identified the area of thin cortical thickness region increased as age grown, and fracture lines were inclined to be more distributed in “B” zone in elderly patients. To meet the demands of acetabular fixation in different age groups, cortical thickness changes in young and elderly individuals should be given special attention when the quadrilateral surface plate is designed.

*Level of evidence* Level IV, observational study.

## Introduction

Management of acetabular fractures is usually challenging due to the inherent difficulties in reducing and fixing the fragments. Operative reduction and stabilization of displaced acetabular fractures are together recognized as the accepted strategy to reduce the risk of traumatic arthritis and realize early mobilization and better functional outcomes. The management of acetabulum fractures, especially osteoporotic fractures, in elderly patients and comminuted fractures in younger patients is difficult [1]. The quadrilateral surface, as the medial wall of the acetabulum, accounted for approximately 10–15% of acetabular fractures, may be damaged easily with less force because of its relatively minimal bone stock [2]. Furthermore, the integrity of the quadrilateral surface is essential to maintain the stability of the acetabulum. Despite progress in treatment strategy during the past few years, it is still challenging to manage quadrilateral surface fractures.

Among many methods to fix the quadrilateral region, an increasingly popular one is the anterior intrapelvic approach, which allows access to the anterior column and medial surface of the posterior column for buttress plate fixation of both columns and offers the benefit of less surgical exposure [3]. However, standard implants such as constructed plates may not be able to obtain satisfactory fixation for displaced quadrilateral regions, so newer preshaped fixation plates have been proposed, with some of these already in use [4]. We also reported that satisfactory fixation can be obtained by using an ‘anatomic quadrilateral surface plate (AQSP) [5]. The advantage of these plates is that repeated precontouring was avoided, and the bottom of the bowl (acetabulum) could be fixed directly through the Stoppa approach [6].

However, to maximize the buttress effect of the AQSP on the quadrilateral surface, the purchase strength of the screws, especially those inserted in combined holes, should be considered, especially in elderly osteoporotic bones. With relatively thinner cortical thickness, the quadrilateral surface lacks enough mechanical strength for stable anchorage of plates or screws, affecting the primary stability. If screws are inserted around the quadrilateral region, which has a relatively weak purchase, protrusion will ensue in the quadrilateral area, and complications such as screw loosening and secondary displacement may occur. Furthermore, there was not research compared the quadrilateral fracture line distribution in different ages. To improve the rate of fixation success and reduce complications, there is a demand to identify the location of the so called weak region and fracture lines distributions in the quadrilateral surface especially for elderly. The aim of the research is to present morphological characteristics in different age groups, such as the position or area of the quadrilateral region, fracture lines distribution, and propose a revised design plate for elderly patients.

## Patients and methods

In this retrospective study (NCT03026868), anonymous pelvis CT data in our traumatic orthopedics center from January 2016 to December 2019 were evaluated. Ethical approval was obtained from the Regional Ethics Committee of our Hospital, and the study was conducted in accordance with the Declaration of Helsinki. Informed signed consent was obtained from all patients who enrolled in the study.

The inclusion criteria were as follows: (1) clear records of demographic data; (2) complete initial CT imaging data; and (3) patients older than 18 years. The exclusion criteria for the study were as follows: (1) pathological fractures (tumor, etc.) and (2) CT with B70 or slices larger than 2 mm.

The subjects were scanned in a clinical whole-body multidetector computerized tomography machine. The threshold values were optimized in accordance with the density histograms of the specimens. The subjects were divided into different groups based on age: Group I = 18–40 years (31 men and 9 women, 31.3 ± 6.6 years), Group II = 41–60 years (54 men and 17 women, 49.9 ± 5.3 years), and Group III ≥ 61 years (25 men and 13 women, 68.7 ± 6.8 years). There was no statistically significant difference in gender composition (*P* = 0.421) or fracture classifications (Table 1, *P* = 0.362) among the three groups. The comorbidities such as lateral femoral cutaneous nerve, sciatic nerve problem injury, obturator nerve injury and soft tissue infection in each group were also recorded in three groups (Table 2).

**Table 1:** The distribution of fracture classification in different age groups

Group	I	II	III		*P*
Anterior column	6	13	7		
Anterior column and posterior hemitransverse	8	21	11		
Both column	22	25	18		
Posterior column and posterior wall	4	12	2	6.578	0.362

**Table 2:** The distribution of post-operation comorbidities in different age groups

Group	I	II	III
Lateral femoral cutaneous nerve	1	1	2
Sciatic nerve problem injury	0	0	0
Obturator nerve injury	0	1	1
Soft tissue infection	0	0	0

### Quadrilateral cortical thickness map construction

Acetabular fracture with one normal semipelvises, including 110 men (48.6 ± 15.0 years) and 39 women (52.7 ± 14.3 years), was collected to present the morphological characteristics of the quadrilateral region. The series of DICOM images originated from PACS (ICZ, Brno, Czech Rep.) and were subsequently reconstructed to identify the morphology of the quadrilateral surface. The DICOM data were then analyzed with Mimics 21.0 (Materialise, Leuven, Belgium), and the thresholding function was used to predetermine the cortical bone value. Hounsfield units of 226 (minimum) and 1600 (maximum) were used as the threshold of the bone tissue in our research. Bone segments and construction functions were used to obtain three-dimensional images of the pelvis.

The 3D pelvis images were obtained and exported from the Mimics medical workstation. The 3D pelvic images were then exported directly to 3 Matic 12.0 (Materialise, Leuven, Belgium). The cortical thickness analysis tool was used to present the different cortical thickness changes in the quadrilateral surface. The minimum threshold was set at 0.33 mm, and the maximum cortical thickness was 20 mm (the maximum possible pixel size). The comprehensive cortical thickness in the quadrilateral surface region can be illustrated completely from the cortical thickness map with different colors.

### Area measurement of the quadrilateral surface (QSA) and thin cortical thickness/width region (TCWRA)

**Fig. 1:**
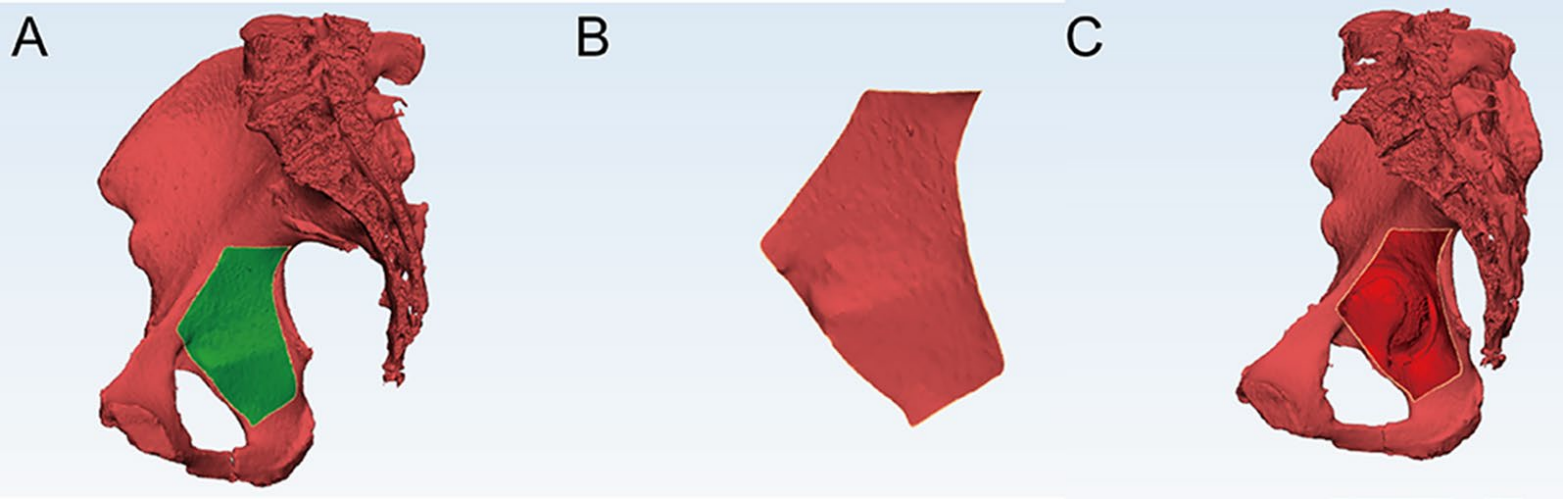
The quadrilateral surface measurement. **a** The green region was identified as a quadrilateral plate, and the area was calculated. **b** The isolated quadrilateral plate. **c** After the plate was deleted, the bottom of the acetabulum was observed

**Fig. 2:**
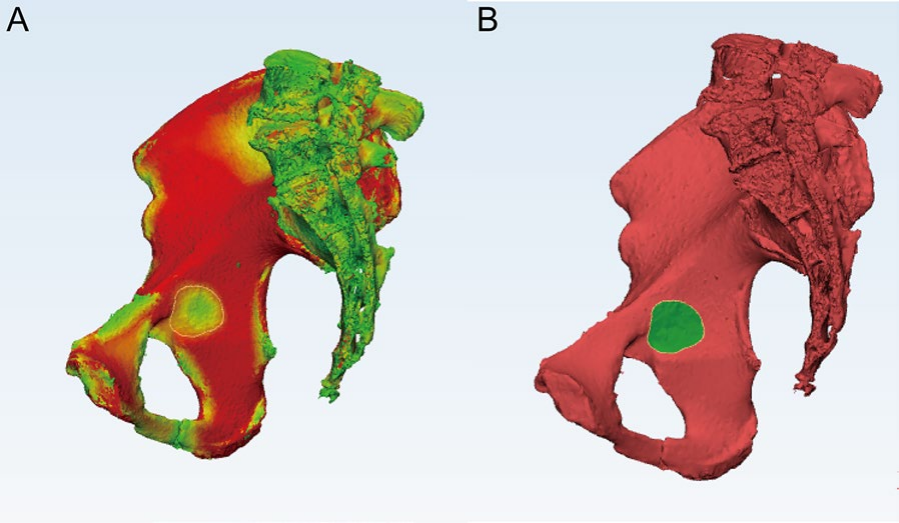
Thin cortical thickness region measurement. **a** The area was circled after the bone thickness analysis was conducted in 3 Matics. **b** The area was illustrated after the bone thickness results were hidden

Curve tools were used to identify the extent of the quadrilateral plate and thin cortical region in the quadrilateral region. The boundary of the quadrilateral region was identified as follows: The upper boundary was the arched line, the lower boundary was the horizontal line of the lower margin of the ischial spine, the anterior boundary was the posterior margin of the obturator foramen and its extension line, and the posterior boundary was the greater sciatic notch and its extension line. A closed curve was formed along the boundary of the quadrilateral plate in 3-dimensional images, and a green region was formed and illustrated (Fig. 1). The thin cortical thickness region was then circled with 3 Matic 12.0 in the cortical thickness map (Fig. 2). Then, the surface area was recorded in the three groups. The ratio of thin cortical thickness regions accounting for the square of the quadrilateral region was also calculated.

**Fig. 3:**
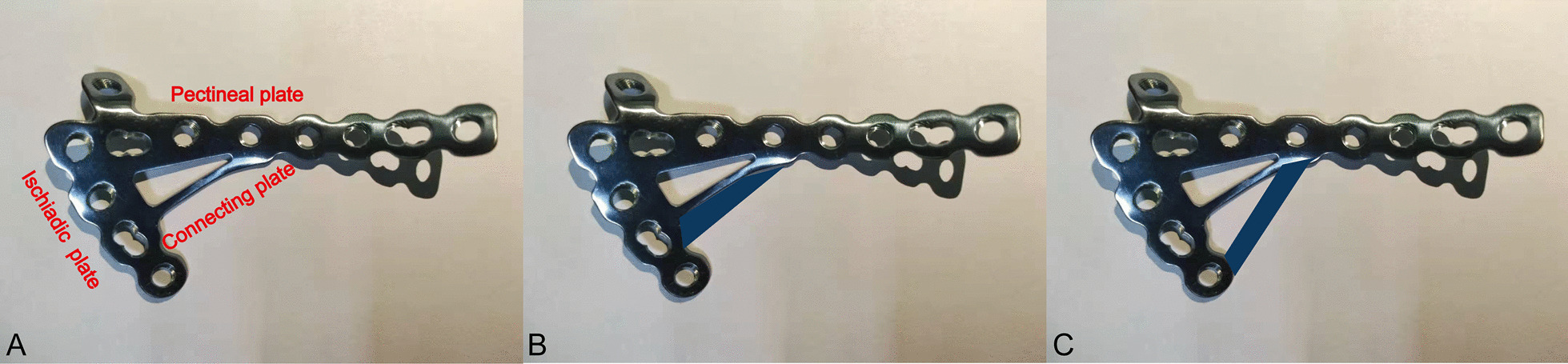
The traditional and newly designed anatomical quadrilateral surface plate. **a** The anatomical quadrilateral surface plate consisted of a pectineal plate, ischiadic plate and connecting plate. **b** The width of the connecting plate can be enlarged to the buttress thin bottom of the acetabulum. **c** The number of connecting plates can be increased to meet different demands, especially in elderly patients

The AQSP consisted of the pubic pectineal, ischiadic part of the plate and connecting plate (CN201610080395, Fig. 3a); it medially spans the anterior and posterior columns through the quadrilateral surface and provides theoretically comparable stability to traditional fixation methods.

### Fracture distribution in 3D map

The fracture lines were simulated freehand onto the templates to demonstrate the quadrilateral fractures in the established template. For ease of analysis, small areas of relatively high comminution fractures were simplified as single fracture lines. To optimize the accuracy of this procedure, the transcriptions were performed by the first authors and were reassessed by an additional trauma surgeons. Any discrepancies between the reviewers were reassessed, and the final results were identified by the corresponding author after a comprehensive evaluation.

### Statistical analysis

Continuous data are illustrated as the means with standard deviation. Homogeneity of variance for continuous variables was evaluated by the Levene test. Comparisons between three age groups were performed by one-way ANOVA with the S–N–K test or Tukey’s test to determine the statistically significant differences between different age groups. For all analyses in this research, significance was set at the *P* < 0.05 level. All analyses were conducted using SPSS Version 26.0 (IBM Corp, Armonk, NY). Spearman correlations were calculated, and linear or nonlinear correlation coefficients were used and calculated to assess the correlation between ages and measurement results. The following correlation parameters were established. When |*r*|≥ 0.8, a high correlation was noted between two variables. For 0.5 ≤|*r*|< 0.8, a moderate correlation was noted. For 0.3 ≤|*r*|< 0.5, a low correlation was noted. When |*r*|< 0.3, there was no correlation.

## Results

### Measurement of the area

**Fig. 4:**
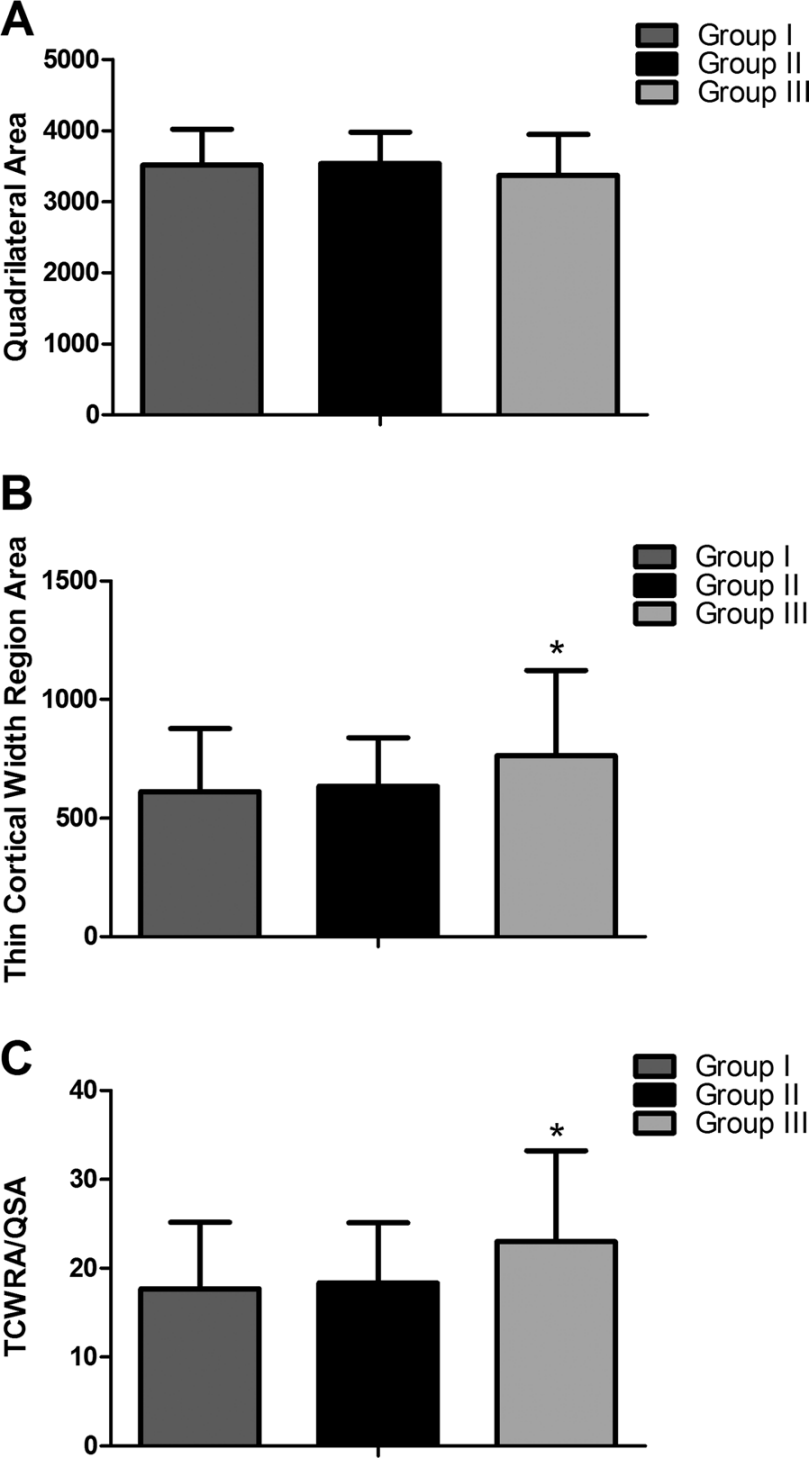
Measurement results. **a** quadrilateral surface area in three groups. **b** The thin cortical thickness region area in the three groups. **c** The ratio was presented in the figures

**Fig. 5:**
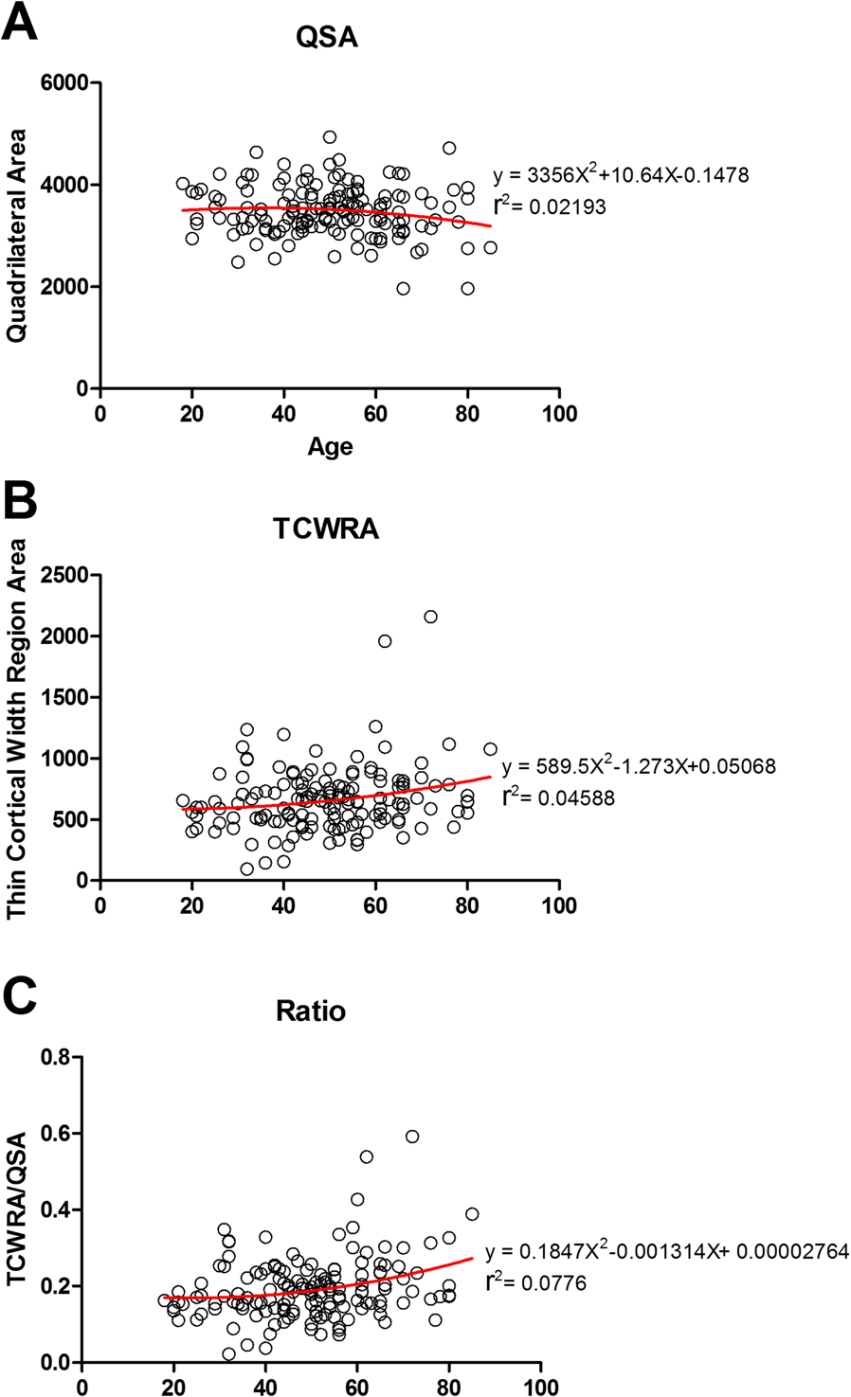
The nonlinear regression equation in three different measurements of all ages. **a** quadrilateral surface area measurement results at all ages. **b** The thin cortical thickness region area measurement results at all ages. **c** The ratio results in all ages

There were no significant differences among the three groups in the total area of the quadrilateral region (Fig. 4a). Age did not predict quadrilateral surface area with *r* = − 0.124, and the nonlinear correlation equation was as follows: *y* = 3356*X*^2^ + 10.64*X* − 0.1478 (Fig. 5A) (Table 3).

**Table 3:** Cortical surface area in quadrilateral, thin cortical thickness region for different age groups [mean (95% CI)]

Groups (m/f)	QSA (mm^2^)	TCTRA (mm^2^)	Ratio (%)
18–40 (m = 31; f = 9)	3512.65 (3351.42, 3673.87)	613.53 (529.20, 697.87)	17.67 (15.26, 20.08)
41–60 (m = 54; f = 17)	3537.25 (3433.60, 3640.90)	635.59 (587.74, 683.44)	18.37 (16.77, 19.96)
Above 60 (m = 25; f = 13)	3367.04 (3177.46, 3556.61)	763.78 (645.57, 881.98)	23.03 (19.68, 26.38)

*QSA* means quadrilateral surface area, *TCTRA* means thin cortical thickness region area, *Ratio* TCTRA/QSA

**Fig. 6:**
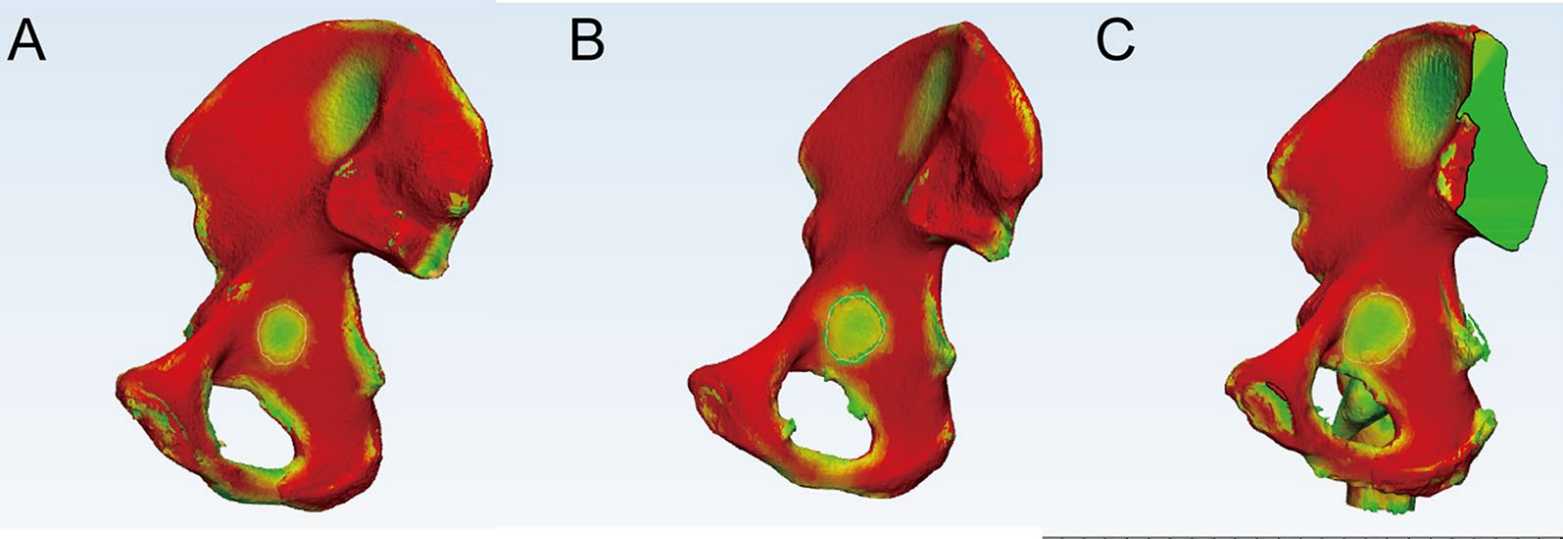
Illustration of the thin cortical thickness in the three age groups

Compared with Group I, the thin cortical thickness/width region area (TCWRA) was significantly increased by 150.25 mm (CI 645.57 to 881.98, + 24.49%) (*P* = 0.037) in Group III (≥ 61 years). Compared with Group II, the TWTRA was also significantly increased by 128.19 mm (CI 645.57 to 881.98, + 20.17%) (*P* = 0.047) in Group III (≥ 61 years). Compared with Group I, no significant differences were found in the TCWRA compared with that of group II (*P* = 0.908) (Fig. 4b). Age did not predict the TCWRA with *r* = 0.2080, and the nonlinear correlation equation was as follows: *y* = 589.5*X*^2^ − 1.273*X* + 0.05068 (Figs. 5b and 6) (Table 3).

Compared with Group I, the ratio of the TCWRA accounted for in the quadrilateral region was significantly increased by 5.36% (CI 19.68 to 26.38, + 30.33%) (*P* = 0.01) in Group III (≥ 61 years). Compared with Group II, the ratio of TCWRA accounted for in the quadrilateral region was also significantly increased by 4.66% (CI 19.68–26.38, + 25.39%) (*P* = 0.011) in Group III (≥ 61 years) (Fig. 4c). Age did not predict the ratio with *r* = 0.2629, and the nonlinear correlation equation was as follows: *y* = 0.1847*X*^2^ − 0.001314*X* + 0.00002764 (Fig. 5C).

### Thin cortical thickness region position distribution

**Fig. 7:**
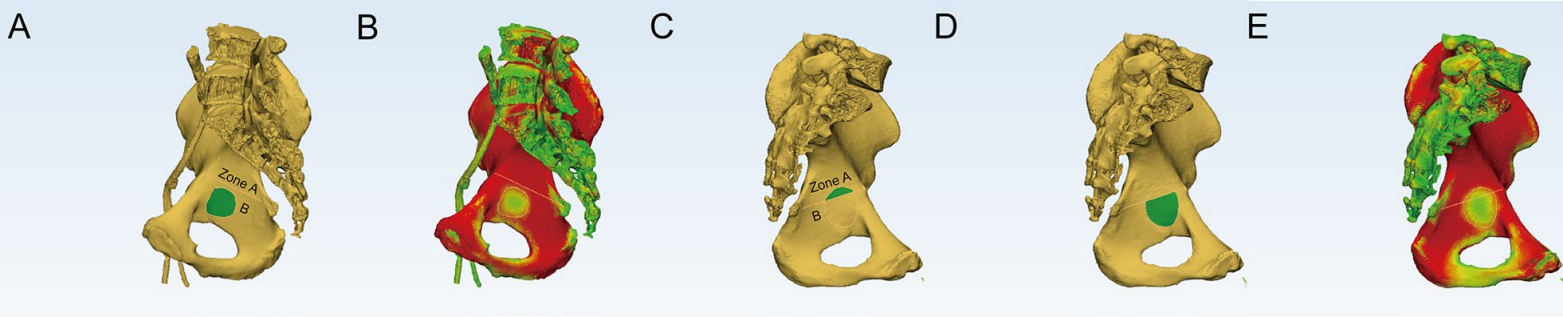
Thin cortical thickness position distribution of the two different types. **a** The region was located in zone “B”. **b** The corresponding cortical thickness map. **c** The region was located in the “A + B” zone, and the small part was in zone “A”. **d** The large part was in zone “B”. **e** The corresponding cortical thickness map in this patient

The line connecting ischial spine and iliopubic eminence divided the quadrilateral plate, and two parts were formed: the rear aspect was named as A zone and the anterior aspect was names as B zone (Fig. 7). None of the subjects had a component involving the “A” zone, thirty-three thin cortical thickness regions (22.15%) were located in the “B” zone, and one hundred and ten–six (77.85%) involved both zones of the quadrilateral plate (“A + B” zone) in the sagittal direction (Fig. 7).

Seven patients were identified whose thin cortical thickness region was located in the “B” zone in Group I, 16 in Group II and 10 in Group III. Thirty-three patients were found in group I, 55 in Group II and 28 in Group III, for whom thin cortical thickness regions were located in the “A + B” zone. There were no significant differences in the position distribution of the thin cortical thickness region in the three different age groups (Table 4).

**Table 4:** The distribution of the thin cortical region in different age groups

Group	“A + B” zone	“B” zone		P
I	33	7		
II	55	16		
III	28	10		
			0.89	0.641

### The fracture maps in three groups

**Fig. 8:**
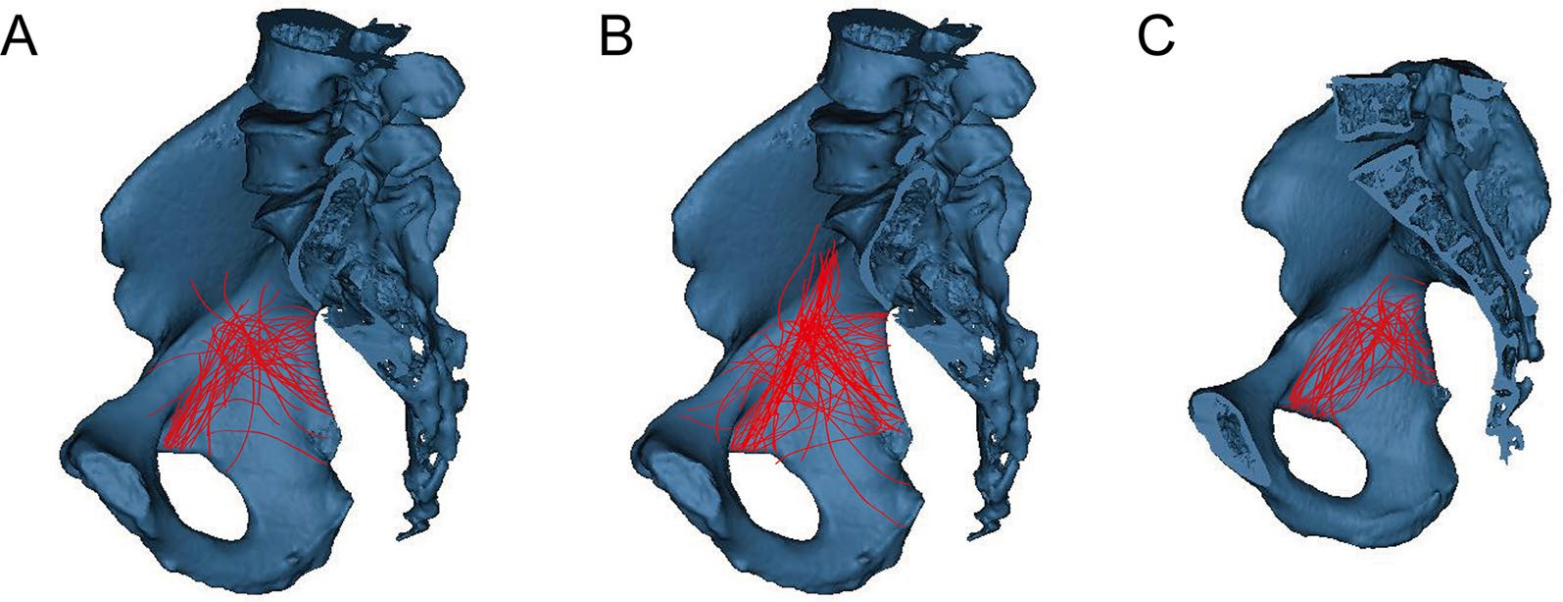
The fracture line distribution in three groups. **a** The fracture lines were mainly focused under the arcuate line. **b** The fracture lines were mainly focused under the arcuate line. **c** The fracture lines were dispersedly distributed under the arcuate line, and adjacent to the bottom of acetabular

The fracture lines of quadrilateral region were mainly and intensively distributed under the arcuate line in Group I and II, and then run obliquely downward to the ischial spine. However, the fractures lines in Group III were also under the arcuate line, but the extent of the fracture lines distribution were enlarged, and more adjacent to the bottom of the acetabular upon the quadrilateral surface (Fig. 8). Twenty-three of the subjects had a component involving the “A” zone (15%), thirty-three thin cortical thickness regions (22%) were located in the “B” zone, and ninety-two (62%) involved both zones of the quadrilateral plate (“A + B” zone) in the sagittal direction (Fig. 8).

Ten patients were identified whose fracture line distribution was located in the “B” zone in Group I, 6 in Group II and 7 in Group III. Eight patients were identified whose fracture line distribution was located in the “B” zone in Group I, 12 in Group II and 13 in Group III. Twenty-two patients were found in group I, 52 in Group II and 18 in Group III, for whom fracture line distribution was located in the “A + B” zone. There were a significant differences of the fracture line distributions in three age groups (Table 5).

**Table 5:** The distribution of the fracture lines in different age groups

Group	“A” zone	“A + B” zone	“B” zone		*P*
I	10	22	8		
II	6	52	12		
III	7	18	13		
				22.336	< 0.001

## Discussion

The purpose of acetabular quadrilateral fracture reduction and fixation is to realize early mobility and mitigate the potential for posttraumatic arthritis by anatomically reducing the articular surface. To improve the rate of internal fixation success, anatomic character of the quadrilateral surface and fracture line distributions in different ages should be known in advance. The research found that the TCWRA increased with aging and mainly located at “A + B” zone with others in B zone in three groups (Figs. 1 and 6). Furthermore, the fracture line distributions were also found to be different between young and elderly patients and more fractures located at anterior parts (B zone) in elderly patients, which mean the bottom of the acetabular was more involved. The AQSP, which we invented, could be adjusted based on those results to meet different clinical demands and to make sure more fractured bottom surface of the acetabulum buttressed in elderly patients. This research promotes our comprehensive understanding of the quadrilateral region and improves the rationality of plate fixation to the quadrilateral surface.

Quadrilateral plate fractures are a heterogeneous group of acetabular fractures that are not formally included in the traditional acetabular classification. The quadrilateral plate, as a thin bony structure, is located on the inner surface of the acetabulum and is adjacent to a number of nerves and blood vessels, such as corona mortis. Furthermore, there is no other bony structure to prevent medial subluxation of the femoral head, so the detached medial surface of the acetabulum and medial migration of the quadrilateral plate with femoral central fracture dislocation are commonly observed when acetabular fractures occur [7–9]. Successful restoration of the buttressing function of the medial wall and prevention of protrusion of the femoral head with internal fixation are essential for early mobilization and improved functional outcomes [10]. Therefore, the maintained reduction and buttress provided by internal fixation to the quadrilateral region are essential. Recently, many newly designed prebent suprapectineal or infrapectineal plates have been proposed to buttress the quadrilateral area which was known as a thin cortical region [11–15]. Furthermore, He found and reported that there was a “safe zone” for infrapectineal plate fixation for quadrilateral surfaces, but he did not mention the effect of screw purchase and fixation construct buttress for the thin part of the quadrilateral surface [16]. In our opinion, the screw should be inserted into the thick cortices to maximize screw purchase. Therefore, it is believed that only an efficient buttress to this thin cortical thickness region and a clearly comprehension of fracture line distributions can truly improve the success rate of quadrilateral fractures fixation.

The plate fixation construct on the quadrilateral surface is challenging not only because of its structure lying directly medial to the acetabulum, but also determined by the inherent characteristics of this area. Although most surgeon known the truth of the existence of the thin cortical region, no research was conducted to explore the more detailed anatomic features of this region, which was so important before new plate designed. The reason for the important of the region was that to realize the maximum purchase and buttress effect, the screw should be inserted in thick cortical bone as close to the thin cortical thickness region as possible, and the area of buttress to the quadrilateral region with plate should be as large as possible. In elder pelvises (≥ 61 years), it was demonstrated that although the quadrilateral surface area, TCWR position, was not significantly changed in different age groups, there was a significantly increased about TCWRA or the ratio of the TCWRA accounted for in the quadrilateral region compared with that of young pelvises (18–60 years), so the buttress needed provided by the anatomical plate was relatively large in elderly patients. Therefore, the types, numbers of screws are definitely restricted, to further improve the rate of fixation, attention on fracture line distribution should also be paid for them.

The number of elderly osteoporotic acetabulum fractures reporting as the most rapidly growing subgroup displays a different distribution of fracture patterns and fracture characteristics than those seen in the younger population and presents a unique set of medical and surgical challenges [8, 17, 18]. To find the character of acetabular fracture line distribution, Yang enrolled 238 quadrilateral fractures and reported that 65% of quadrilateral fractures involved both rear and anterior parts, which was comparable with ours (62%) [17]. Furthermore, he also reported that only 10% of quadrilateral fractures involve anterior parts (B zone) which was significantly different with ours (22%). It was found that as age increases, an increasing number of fractures will involve the anterior parts (B zone) in Group III. The reason for the differences can be explained as that most enrolled patients were young with an average age of 43 years in his research, which did not represent the entire age groups especially for the elderly. The result about the thin cortical thickness region position distribution and ratio of TCWRA accounted for in the quadrilateral region was another evidence to reveal the reason for differences of fracture line distributions that the area of the region was enlarged in aged patients, which meant that more bone stock was lost around the bottom of acetabular. Therefore, fixation in older patients should be more meticulous, and the area buttress to the quadrilateral region should be enlarged. To meet the demand, the plate used in the elderly pelvis can be revised, and the connecting plate can be widened or another connecting plate can be added that connects the pectineal and ischiadic parts of the AQSP (Fig. 3b, c). Chen compared different fixation techniques for typical acetabular fractures in the elderly and concluded that special infrapectineal quadrilateral surface buttress plates provided stiffness and stability comparable with the standard fixation of suprapectineal pelvic brim plates with 3 periarticular long screws [13]. The AQSP was classified as an infrapectineal buttress plate, and the specially designed hole that fixes the anterior and posterior columns can further strengthen the fixation stiffness compared with the quadrilateral surface buttress plate designed by others. Therefore, under the guidance of anatomic results and fracture line distribution about quadrilateral plate found in this research, it was believed that the modified AQSP can further improve the fixation effect efficiently in elderly quadrilateral fractures, and relevant mechanical experiment will also be conducted in our following research.

The limitation of the article was that the cortical thickness analysis was conducted with bone segment function, and whether the results were equal to the split function in Mimics was not tested. Second, the relationship or difference between the thin cortical thickness region proposed by us and the safe zone proposed by other researchers was not analyzed, but the main topic in this research was to differentiate the buttress effect needed by AQSP in young and elderly individuals, not how to avoid screw invasion of the acetabulum. Third, the type of injury was not collected in this research, and more elderly patients might be injured by low energy, but what we considered in this study was the fracture line distribution and treatment strategy upgrade for them, so the injury mechanism will have limited effect on the research results. At last, the female patients and patients in Group III enrolled was limited, and more orthopedic departments will be united to clarify the fracture line distribution differences between male and female patients in our following research.

## Conclusion

In conclusion, this research identified the area of thin cortical thickness region increased as age grown. The fracture lines were more inclined to approach the bottom of the acetabular (anterior parts, B zone) in elder people. To meet the fixation demands of different age groups, cortical thickness changes in young and elderly individuals should be given special attention when the quadrilateral surface plate is used and designed.

## Data Availability

All the data were contained in the article.
